# *Salmonella* Dublin outbreaks in Brazilian cattle: clinical-epidemiological aspects, antimicrobial resistance, and comparative genomic analysis

**DOI:** 10.1128/spectrum.02665-25

**Published:** 2026-03-12

**Authors:** Isabela Pádua Zanon, Victor Santos do Amarante, Sophia Kenney, Tales Fernando da Silva, Yasmin Gonçalves de Castro, João Victor Ferreira Campos, Vasco Azevedo, José Azael Zambrano Uribe, Fabrício Gomes Melo, Flávia Figueira Aburjaile, Bertram Brenig, Henrique Cesar Pereira Figueiredo, Erika Ganda, Rodrigo Otávio Silveira Silva

**Affiliations:** 1 Escola de Veterinária, Universidade Federal de Minas Gerais (UFMG) 28114, Belo Horizonte, Brazil; 2 Department of Animal Science and One Health Microbiome Center, Pennsylvania State University 8082 , University Park, Pennsylvania, USA; 3 Instituto de Ciências Biológicas (ICB), Universidade Federal de Minas Gerais (UFMG) 28114, Belo Horizonte, Brazil; 4 Institute of Veterinary Medicine, University of Göttingen 9375, Göttingen, Germany; Earlham Institute, Norwich, United Kingdom

**Keywords:** salmonellosis, zoonosis, livestock, pneumonia

## Abstract

*Salmonella enterica* serotype Dublin is a major concern for livestock, particularly due to its high morbidity and mortality rates in cattle herds. In Brazil, limited research has explored the epidemiology of this serovar, including outbreak characterization, antimicrobial susceptibility profiles, and genetic similarity of isolates. This study aimed to describe the clinical and epidemiological features of confirmed *S.* Dublin outbreaks across multiple Brazilian cattle herds, as well as to assess antimicrobial resistance profiles, identify resistance genes, and evaluate genetic similarity among isolates. Between 2018 and 2025, *S.* Dublin outbreaks were confirmed on 19 farms, most of which (84.2%) were dairy facilities involving calves. In 57.9% of the outbreaks, calves aged 90–180 days were affected. Notably, 68.4% of the farms reported *S.* Dublin outbreaks following or coinciding with an increase in cases of bovine tick fever (babesiosis and/or anaplasmosis). The majority of isolates (79.5%) were resistant to at least one antimicrobial, and 20.5% were classified as multidrug-resistant. Among the resistance determinants identified, a point mutation in gyrA, detected in more than a quarter of the sequenced isolates, was particularly notable. Single-nucleotide polymorphism (SNP) and core genome multilocus sequence typing (cgMLST) analyses revealed marked genetic similarity among isolates from related outbreaks, highlighting the risk of introducing *S.* Dublin through the purchase of untested animals. A high similarity was also observed between outbreak isolates and strains previously associated with human infections in Brazil, underscoring the zoonotic potential of *S.* Dublin.

**IMPORTANCE:**

*Salmonella* Dublin is a cattle-adapted serovar capable of causing disease and substantial economic losses in the livestock sector. In humans, this serotype is also associated with serious illness and has the highest reported fatality rate among *Salmonella enterica* serotypes. In this study, we aimed to describe 19 outbreaks of *S.* Dublin in cattle herds and to characterize the clinical and epidemiological features of the disease in cattle, given the scarcity of information in the literature. Alongside this, we sequenced at least one isolate per farm to investigate antimicrobial resistance genes, mobile genetic elements, and the similarity between our strains and those from bovine and human sources in Brazil and elsewhere. Overall, this is the first study to assess the clinical aspects of *S.* Dublin in cattle herds from tropical regions, accompanied by the antimicrobial profiles of these isolates and their genomic relatedness to strains isolated from human clinical infections.

## INTRODUCTION

*Salmonella enterica* serovar Dublin is a bovine-adapted serotype capable of causing a range of clinical outcomes, including outbreaks with high morbidity and mortality, leading to considerable economic losses for livestock farming (1–3). Among adult animals, the disease is associated with abortions in the last third of pregnancy and decreased milk production (2, 4, 5). In calves, this serovar frequently causes acute infection, often presenting with respiratory signs, enteric disease, and, in many cases, septicemia (6–8). It is important to note that this serovar may result in chronic asymptomatic carriers, who contribute bacterial shedding into the environment (8–10).

Despite the recognized importance of *S*. Dublin in cattle, its epidemiology remains poorly understood, particularly in Latin American countries (11). In Brazil, only one study conducted by our research group has investigated the shedding dynamics of *S*. Dublin during an outbreak in a cattle herd on a farm in the Central-West region (12). In contrast, the remaining studies are mostly limited to case reports (13–15). In addition, epidemiological studies conducted in other countries have focused on indirect diagnostic approaches, especially the detection of antibodies in milk, and have been limited to temperate-climate regions (16–20). Thus, the clinical and epidemiological characteristics of *S*. Dublin infection in cattle raised under tropical production systems remain largely unexplored.

In addition to its importance in livestock, *S*. Dublin is also a major serotype linked to invasive and severe infections in humans (21–24). Interestingly, this serotype has been associated with higher lethality compared to other serotypes, frequently resulting in extended hospitalization and increased mortality (22, 25, 26). In this context, recent studies have demonstrated a high genetic similarity between *S*. Dublin isolates from infected animals and humans, supporting the hypothesis that production animals serve as significant reservoirs of *Salmonella* sp. (11, 12, 27–31). Consequently, the presence of antimicrobial resistance in *S*. Dublin isolates from cattle raises serious concerns, as it may considerably hinder effective treatment of infected patients, especially those in high-risk groups (32–34). Reflecting this threat, the World Health Organization has classified fluoroquinolone-resistant *Salmonella* spp. as a high-priority pathogen for the development of new antimicrobial agents (35).

Despite the increasing significance of *S*. Dublin in cattle production and its notable zoonotic impact, especially regarding multidrug-resistant isolates, few studies have evaluated strains of this serotype in Latin American countries, including Brazil. Moreover, the associated clinical manifestations, affected animal categories, and antimicrobial resistance determinants involved in the disease epidemiology remain largely uncharacterized. Therefore, the present study aimed to describe the clinical and epidemiological characteristics of confirmed *S*. Dublin outbreaks across multiple farms in Brazil, in addition to characterizing antimicrobial resistance profiles, identifying resistance determinants, and evaluating the genetic relatedness of the isolates.

## MATERIALS AND METHODS

### Sample selection and metadata collected

**Fig 1:**
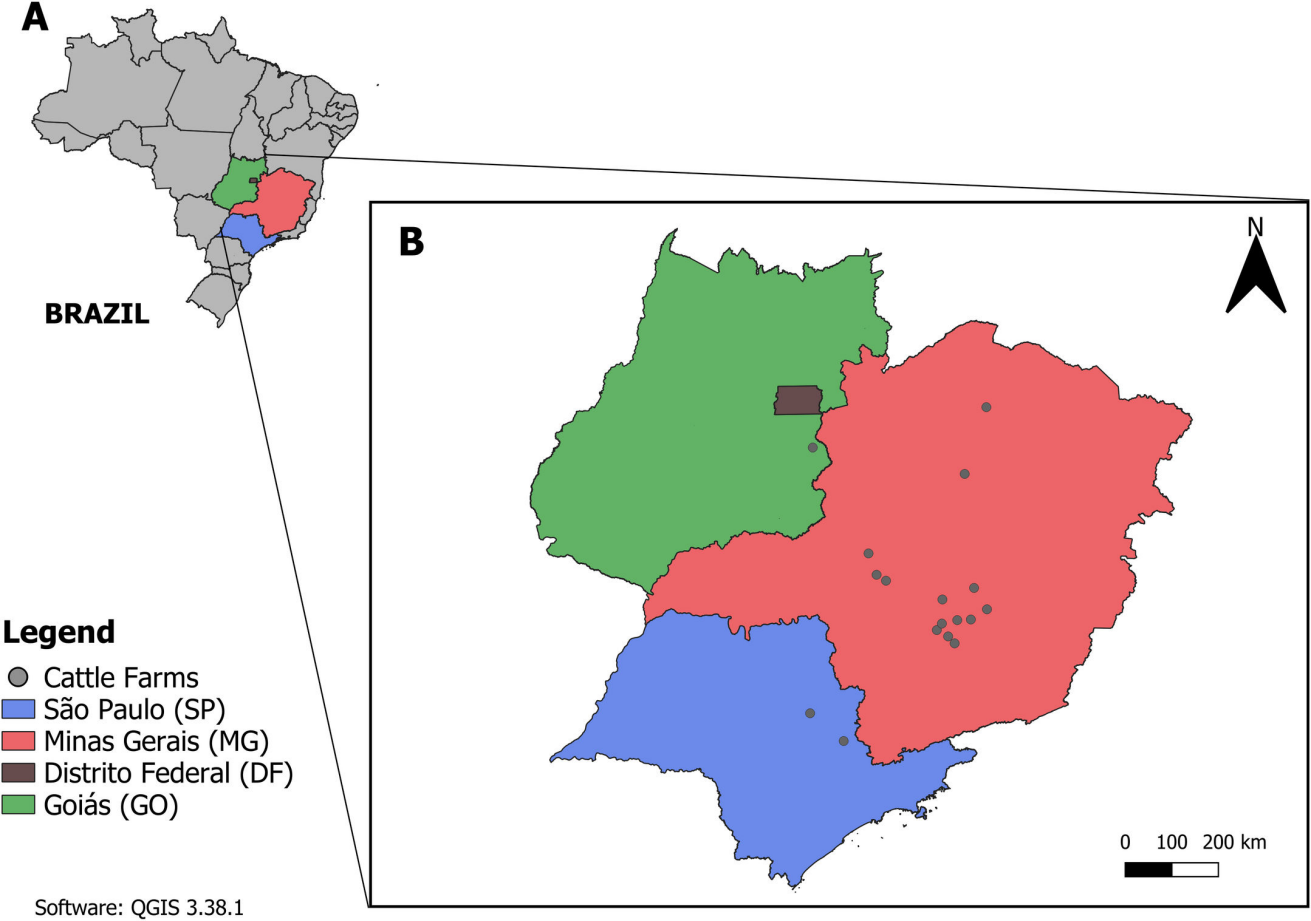
Map showing the location of cattle herds with confirmed outbreaks of *S*. Dublin. (**A**) Map of Brazil showing the location of the states: Goiás, Minas Gerais, and São Paulo. (**B**) Enlarged view of panel A, highlighting the states where the affected herds are located.

A total of 44 isolates from 19 farms in Brazil that experienced *S*. Dublin outbreaks between 2018 and 2025 were included in the study. These strains originated from three Brazilian states, with 16 farms located in Minas Gerais, two in São Paulo, and one in Goiás (Fig. 1). The following data related to each outbreak were collected and analyzed when available: farm location, history of animal purchases and sales, average daily milk production, age and clinical signs of the affected animals, post-mortem findings, and presence of comorbidities (File S1). In four farms (cases 7, 12, 16, and 18), samples from liver, lung, gallbladder, spleen, small intestine, and kidney were collected for histopathological analysis.

### Isolation and characterization of *Salmonella* Dublin

Samples from extraintestinal organs (lung, heart, spleen, liver, brain, gallbladder, and ocular conjunctiva, *n* = 39) and intestinal contents (*n* = 5) of necropsied animals suspected of *Salmonella* infection were plated on Hektoen Enteric agar (Oxoid, USA) and MacConkey agar (Difco, USA), followed by incubation under aerobic conditions at 37°C for 18 to 24 h. Simultaneously, samples were pre-enriched in Rappaport-Vassiliadis broth (Merck, Germany) at 37°C for 24 h, followed by plating on Hektoen Enteric agar (Oxoid, USA) and MacConkey agar (Difco, USA). Isolates were then subjected to DNA extraction (36) and genus-specific polymerase chain reaction (37). Strains confirmed as *Salmonella* sp. were serotyped by antigenic characterization according to the White-Kauffmann-Le Minor scheme (38).

### Antimicrobial susceptibility of *Salmonella* Dublin isolates

The antimicrobial susceptibility of the isolates was tested using the agar disk diffusion method, as previously described by the Clinical and Laboratory Standards Institute documents, M100-Ed31 (39), and VET01-Ed6 (40). Fourteen antimicrobials representing 11 different classes were tested: amikacin (30 μg), amoxicillin/clavulanic acid (30 μg), ampicillin (10 μg), azithromycin (15 μg), cephalothin (30 μg), ceftriaxone (30 μg), ciprofloxacin (5 μg), chloramphenicol (30 μg), gentamicin (10 μg), fosfomycin (200 μg), meropenem (10 μg), nitrofurantoin (300 μg), sulfamethoxazole-trimethoprim (25 μg), and tetracycline (30 μg). *Escherichia coli* ATCC 25922 was used as a control. Isolates resistant to three or more antimicrobial classes were classified as multidrug-resistant (41).

### Whole-genome sequencing and comparative genomics

At least one isolate from each farm was subjected to whole-genome sequencing (*n* = 22 strains) (File S2). For DNA extraction, each isolate was plated on Mueller–Hinton agar (Kasvi, Brazil) at 37°C for 24 h. Genomic DNA was extracted using the kit Wizard Genomic DNA Purification (Promega, EUA). Genome sequencing was performed using the Illumina MiSeq platform (mid-out 2 × 150 bp cycles). The raw data were analyzed using FastQC (Babraham Bioinformatics, Cambridge, England), retaining only paired reads with a Phred quality of 30 or higher and a minimal size of 50 nucleotides.

The assembly was performed using SPAdes 3.5.0 in the careful mode (42, 43). GAP filling and polishing were performed using Pilon (44). The genomes have been deposited in the National Center for Biotechnology Information (NCBI) under BioProject accession numbers PRJNA1133727 and PRJNA1150938.

ResFinder 4.5 (45, 46) and MobileElementFinder v1.0.3 (47) were used with default parameters to identify acquired antimicrobial resistance determinants and mobile genetic elements, respectively. PubMLST (48) was used to determine sequencing types. Core genome multilocus sequence typing (cgMLST) was performed using the Galaxy @Sciensano (49) platform (https://galaxy.sciensano.be), applying the *Salmonella enterica* cgMLST scheme from EnteroBase. Allele calling was carried out using a BLAST-based approach, and phylogenetic relationships were inferred using the MSTreeV2 algorithm. Only loci detected in at least 90% of the analyzed genomes were retained. Allelic profiles were analyzed using GrapeTree software (50) to generate minimum spanning trees. A cut-off value of 10 allelic differences was applied to define clusters of closely related strains (30).

Single-nucleotide polymorphisms (SNPs) were called from whole-genome sequence data using CSIPhylogeny (51, 52), applying a minimum Z-score of 1.96 and a minimum sequencing depth of 10× at SNP positions. The *Salmonella* Dublin ATCC 39184 strain (accession number SAMN01041081) was used as the reference genome for SNP calling, a well-characterized reference from the American Type Culture Collection (ATCC) from the same serovar. This approach was chosen to allow a good visualization of the genetic similarity even between closely related isolates. Brazilian strains of *S*. Dublin from infected cattle (*n* = 10) and humans (*n* = 25) isolated in the last 20 years were obtained from the Bacterial and Viral Bioinformatics Resource Center database (27, 53) and used for comparison (File S2). The phylogenetic tree was visualized using iTOL v.6 online (54) and midpoint rooted. A second tree based on SNP analysis rooted using a *Salmonella* Enteritidis reference genome (accession number AM933172) as an outgroup was also included (File S3). This second analysis was included in order to see ancestral relationships, and the reference strain was chosen based on established serotype-level phylogenetic relationships between *Salmonella* Enteritidis and *Salmonella* Dublin, as previously described (55–57).

To contextualize Brazilian strains in the global *Salmonella* Dublin population, the NCBI Pathogen Detection Browser was used to identify publicly available strains meeting the following inclusion criteria: human or bovine associated, collected between 2018 and 2024, and computed serotype “9:g,p:-.” Stratified by collection year and region, strains from each non-Brazilian country were randomly subsampled to approximately 30 strains per country for bovine strains or to 15 strains per country for human strains. Raw reads were processed through the quality control and assembly pipeline previously described (58). Prokka v1.14.6 (59) was used for genome annotation prior to core genome alignment using MAFFT v7.525 (60) with Roary v3.13.0 (61). A maximum-likelihood phylogeny was generated with IQTree (62) using 1,000 UFBoot (63) bootstrap replicates and the GTR+I+G4 nucleotide substitution model. Results were visualized in RStudio with *ggtree* v3.14.0 (64). All code for this component of the analysis is available at https://github.com/sophiakenney/brazildublin.

### Statistical analysis

Antimicrobial susceptibility data, including resistance to different classes of antibiotics and multidrug resistance profiles, were summarized using frequency tables. Univariate analysis was conducted using Fisher’s exact test to assess associations and calculate odds ratios (OR) between resistance to specific antibiotics and multidrug resistance. All analyses were performed using Stata/SE 12.0 (StataCorp, 2011).

## RESULTS

### Outbreak description

Between 2018 and 2025, 19 farms were diagnosed with outbreaks of *Salmonella* Dublin. Most cases (16/19 = 84.2%) occurred in dairy herds and affected calves, while two outbreaks occurred in a beef cattle feedlot and one in a veterinary hospital. The size of affected dairy farms ranged from 50 to 1,200 lactating cows (Table 1). In most outbreaks (11/19 = 57.9%), calves between 90 and 180 days of age were affected. One outbreak affected lactating cows (1/19 = 5.3%).

**TABLE 1:** Case, year, number of isolates per case, average daily production (L/cow), number of lactating cows, and age of affected animals from each farm^*a*^

Case	Year	Number of isolates	Average daily milk production (L/cow)	Lactating cows	Age of affected animals
Case 1	2019	1	N/A	N/A	3–6 months
Case 2	2019	2	25	100	20 days
Case 3	2019	1	10	70	Lactating cows
Case 4	2019/2020	4	32	890	3–5 months
Case 5	2020	1	Healthcare-associated infection		20–60 days
Case 6	2020	1	12	50	60 days
Case 7	2021	1	36	560	4–6 months
Case 8	2021	12	30	900	2–5 months
Case 9	2021	1	N/A	N/A	20 months
Case 10	2022	1	N/A	N/A	20 months
Case 11	2022	1	31	800	4–6 months
Case 12	2022/2023	2	35	350	3–6 months
Case 13	2022	1	26	40	60 days
Case 14	2022	1	31	270	3 months
Case 15	2022/2023/2025	6	38	1,200	3–5 months
Case 16	2023	1	36	700	3–5 months
Case 17	2024	1	34	140	15–75 days
Case 18	2024	1	35	400	3–5 months
Case 19	2024/2025	5	22	75	10–20 days and 3 to 7 months

^*a*^ N/A, not available.

Reported clinical signs included respiratory distress, apathy, loss of appetite, hyperthermia, and jaundice. Regarding comorbidities, most farms (13/19 = 68.4%) reported that the *S*. Dublin outbreak coincided with an increased incidence of tick fever (babesiosis and/or anaplasmosis). On one farm, lactating cows were simultaneously diagnosed with salmonellosis and trypanosomosis (*Trypanosoma vivax*), presenting with diarrhea and dying with signs of sepsis.

**Fig 2:**
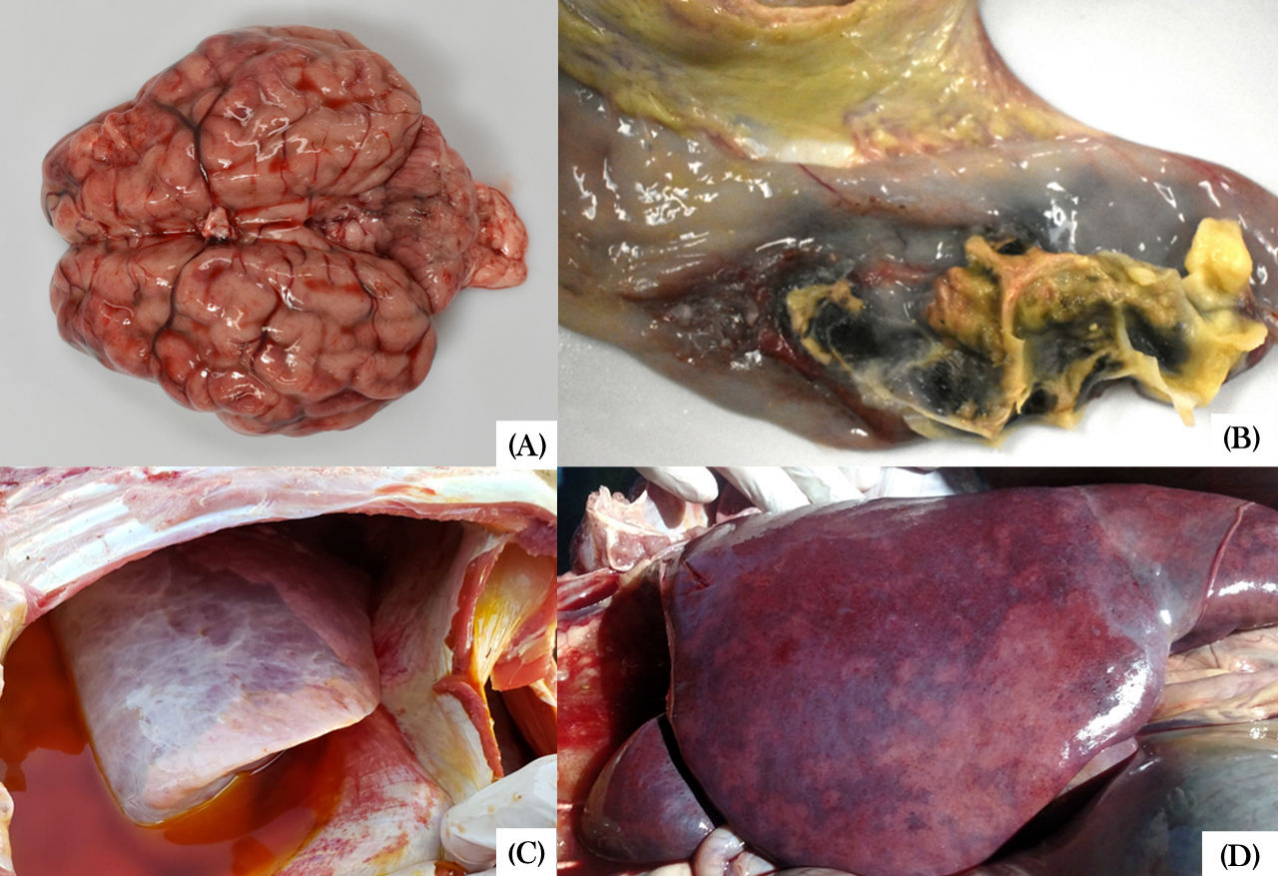
Necropsies of calves with *S*. Dublin isolation. Case 5 (calf aged between 20 and 60 days): brain with marked hyperemia of leptomeningeal vessels (**A**) and typhlitis (**B**). Case 4 (calf aged between 91 and 150 days): ascites (**C**). Case 2 (20-day-old calf): hepatomegaly and irregular coloration on the liver surface (**D**).

**Fig 3:**
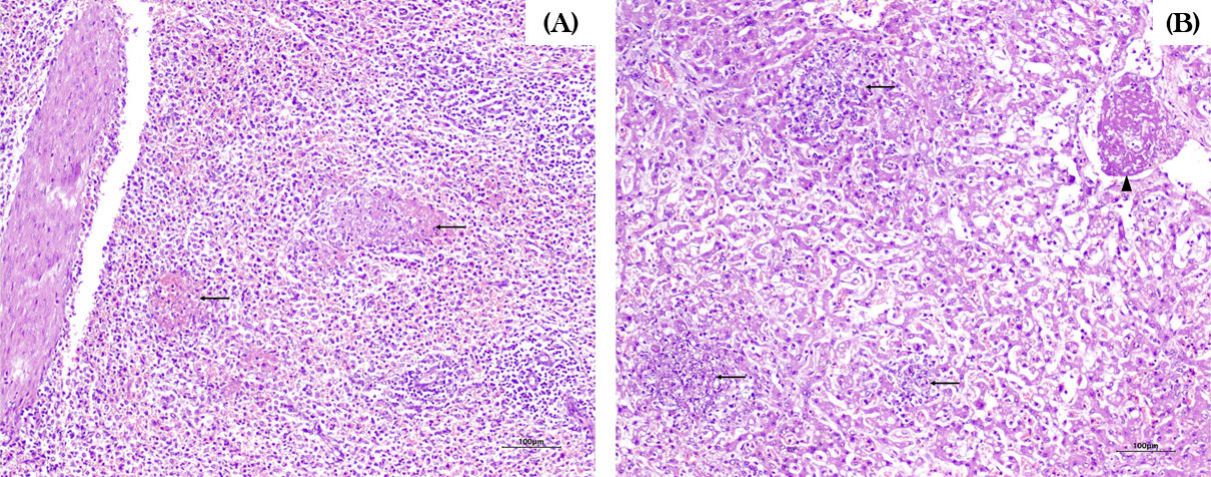
Histopathological findings in cattle herds affected by *S*. Dublin. (**A**) Spleen with areas of necrosis, fibrin deposition, and neutrophilic and histiocytic inflammatory infiltrate (arrows). (**B**) Liver with areas of necrosis and loss of hepatocytes, replaced by fibrin and a predominantly histiocytic inflammatory infiltrate, with occasional neutrophils (arrows). Fibrin thrombi are present within the lumen of some blood vessels (arrowhead).

The most reported post-mortem findings included marked hyperemia of the mucous and serous membranes of the organs, followed by pulmonary edema, petechiae in the oral and vulvar mucosa, hepatomegaly, splenomegaly, and a distended gallbladder containing thick contents (Fig. 2). In one case (case 5), the intestines exhibited hyperemic serosa and yellowish fluid content, consistent with diffuse catarrhal-hemorrhagic enteritis. Also in this outbreak, one of the necropsied animals presented marked vascular hyperemia in the leptomeninges (Fig. 2A), accompanied by an accumulation of yellowish material consistent with suppurative meningitis, as well as fibrillar material deposition in the joints, indicative of fibrinous arthritis. Histopathological findings revealed intense inflammatory infiltrates, predominantly neutrophilic and lymphohistiocytic, with fibrin deposition in multiple organs, including liver, lungs, kidneys, rumen, spleen, and brain. In some cases, intralesional bacterial aggregates with coccobacillary morphology were observed (Fig. 3).

### Antimicrobial susceptibility

**Fig 4:**
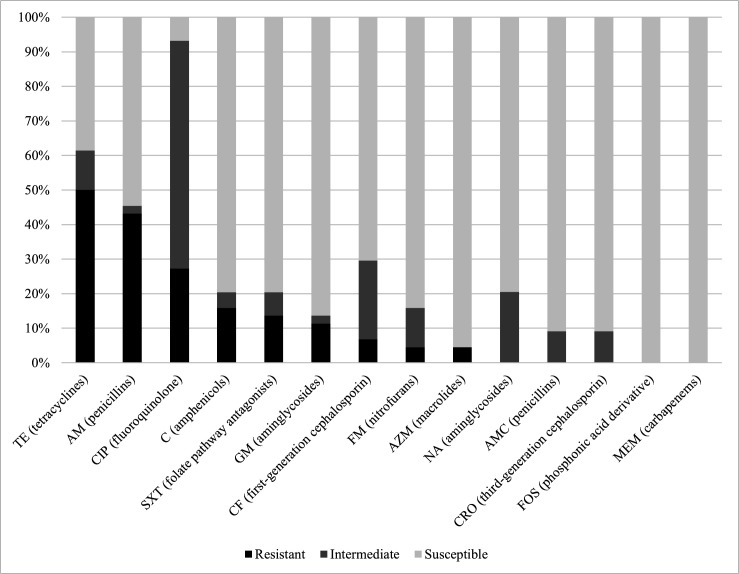
Antimicrobial resistance profile of *S*. Dublin isolates (*n* = 40) from outbreaks in Brazilian cattle between 2018 and 2025. AM, ampicillin; TE, tetracycline; CIP, ciprofloxacin; STX, sulfamethoxazole-trimethoprim; C, chloramphenicol; GM, gentamicin; CF, cephalothin; FM, nitrofurantoin; AN, amikacin; AMC, amoxicillin-clavulanate; AZM, azithromycin; CRO, ceftriaxone; FOS, fosfomycin; MEM, meropenem.

Most isolates (35/44 = 79.5%) were resistant to at least one antimicrobial, and nine (9/44 = 20.5%) were classified as multidrug-resistant. At least one multidrug-resistant isolate was found on 21.1% (4/19) of farms. Half of the isolates were resistant to tetracycline (50.0%), nearly half were resistant to penicillins (43.2%), and more than a quarter of the isolates (12/44 = 27.3%) were resistant to fluoroquinolones (Fig. 4). In the present study, isolates resistant to fluoroquinolones, penicillins, amphenicols, or first-generation cephalosporins were more likely to exhibit a multidrug-resistant profile (*P <* 0.05). In addition, fluoroquinolone-resistant isolates were almost 10 times more likely to be multidrug-resistant (OR, 9.67).

### Comparative genomics

**Fig 5:**
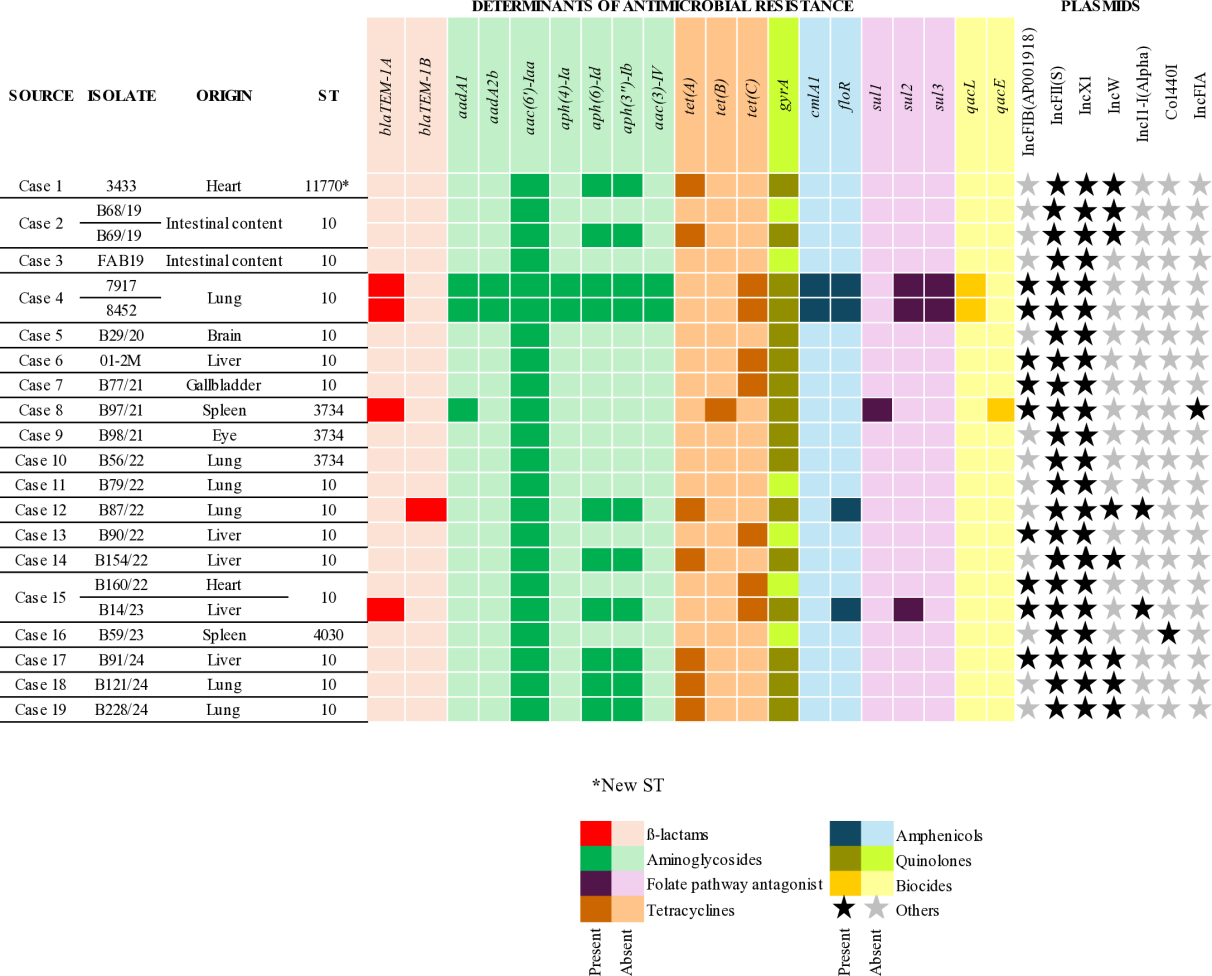
Antimicrobial resistance determinant genes, sequence types (STs), and plasmids identified in *S.* Dublin strains sequenced from each case.

The *aac(6´)-Iaa* gene (22/22; 100%) and point mutation in *gyrA* gene (16/22; 72.7%), conferring resistance to aminoglycosides and fluoroquinolones, respectively, were the most frequently detected antimicrobial resistance determinants (Fig. 5). Two-point mutations in *gyrA* were seen in the present study: S83Y in one isolate (B29/20) and S83F in the remaining strains. These mutations are well-known markers of fluoroquinolone resistance in *Salmonella enterica* and have been previously reported in *S*. Dublin isolates from Brazil (65–70).

Three genes conferring resistance to tetracycline antimicrobials were detected: *tetA* (*n* = 7), *tetC* (*n* = 7), and *tetB* (*n* = 1). The two isolates from case 4 stood out due to the presence of resistance determinants for seven classes of antimicrobials, five of which were detected exclusively in isolates from this case: *aadA2b*, *aph(4)-Ia*, and *aac(3)-IV*, conferring resistance to aminoglycosides; *sul3*, associated with sulfonamide resistance; and *qacL*, conferring resistance to biocides. Isolates harboring resistance determinants for tetracyclines, penicillins, cephalosporins, and amphenicols also exhibited phenotypic resistance, suggesting a strong correlation between genotypic and phenotypic methods.

### Plasmid detection

Seven plasmid replicons were detected in the sequenced *S*. Dublin strains (*n* = 22) (Fig. 5). Plasmid replicons IncFII (S) and IncX1 were identified in all isolates. Analysis of plasmid replicons revealed that some carried antimicrobial resistance determinants (File S4). The *tetA* gene, associated with tetracycline resistance, was located on an IncW plasmid in all isolates where it was detected.

Isolates from case 4 (7917 and 8452) stood out due to the presence of IncFIB(AP001918), which harbored resistance genes associated with amphenicols (*cmlA1*), folate pathway antagonists (*sul3*), aminoglycosides (*aadA1* and *aadA2b*), and biocides (*qacL*). Similarly, an isolate from case 15 (B14/23) carried IncI1-I(Alpha), which also harbored resistance genes related to amphenicols (*floR*), folate pathway antagonists (*sul2*), and aminoglycosides [*aph(6)-Id* and *aph(3'')-Ib*].

### Strain sequence typing and relatedness

MLST analysis revealed that all isolates belonged to clonal complex (CC) 53, with the majority of strains assigned to ST10 (17/22; 77.3%). One novel sequence type (ST11770) was identified in the present study.

**Fig 6:**
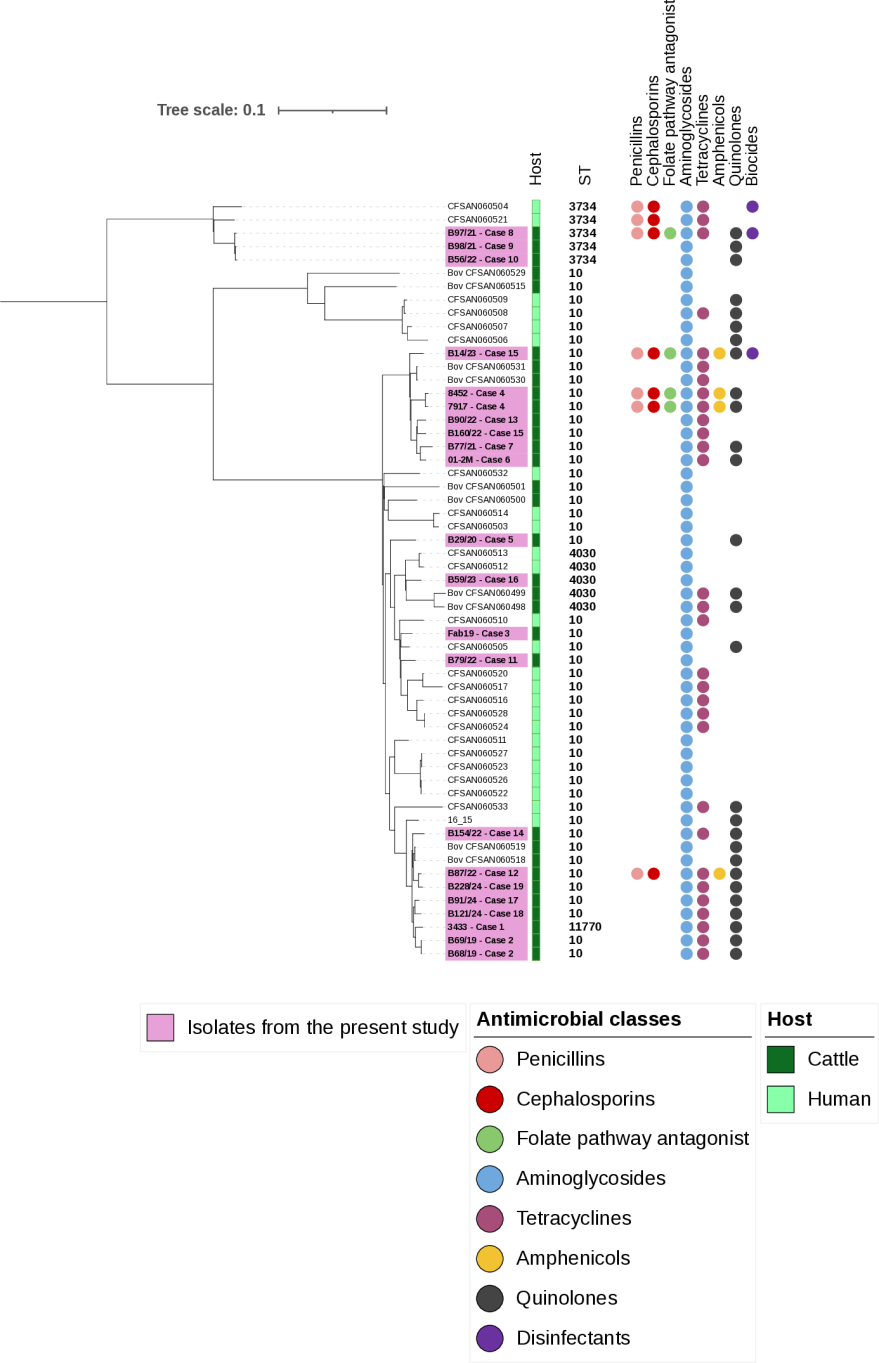
Single-nucleotide polymorphism (SNP) analysis showing the genetic relationship between *S*. Dublin isolates from cattle and humans. *S*. Dublin isolates from previous studies in Brazil (27) were added for comparison. A total of 547 high-quality SNPs were used for tree construction, and the tree visualization was generated using iTOL (54) online, applying midpoint rooting.

SNP analysis revealed a high similarity between strains from the same farm (Fig. 6), such as B68/19 and B69/19 (case 2), and 7917 and 8452 (case 4), which differed by up to eight SNPs (File S5), below the previously established 15-SNP threshold for assessing potential clonality (23, 71, 72). Strain B98/21 (case 9) differs by two and three SNPs from strains B56/22 (case 10) and B97/21 (case 8). Furthermore, strains B56/22 and B97/21 differ by five SNPs (Fig. 6). Isolate B87/22 showed 13 and 14 SNP differences compared with two strains isolated from cattle in a prior study (73).

**Fig 7:**
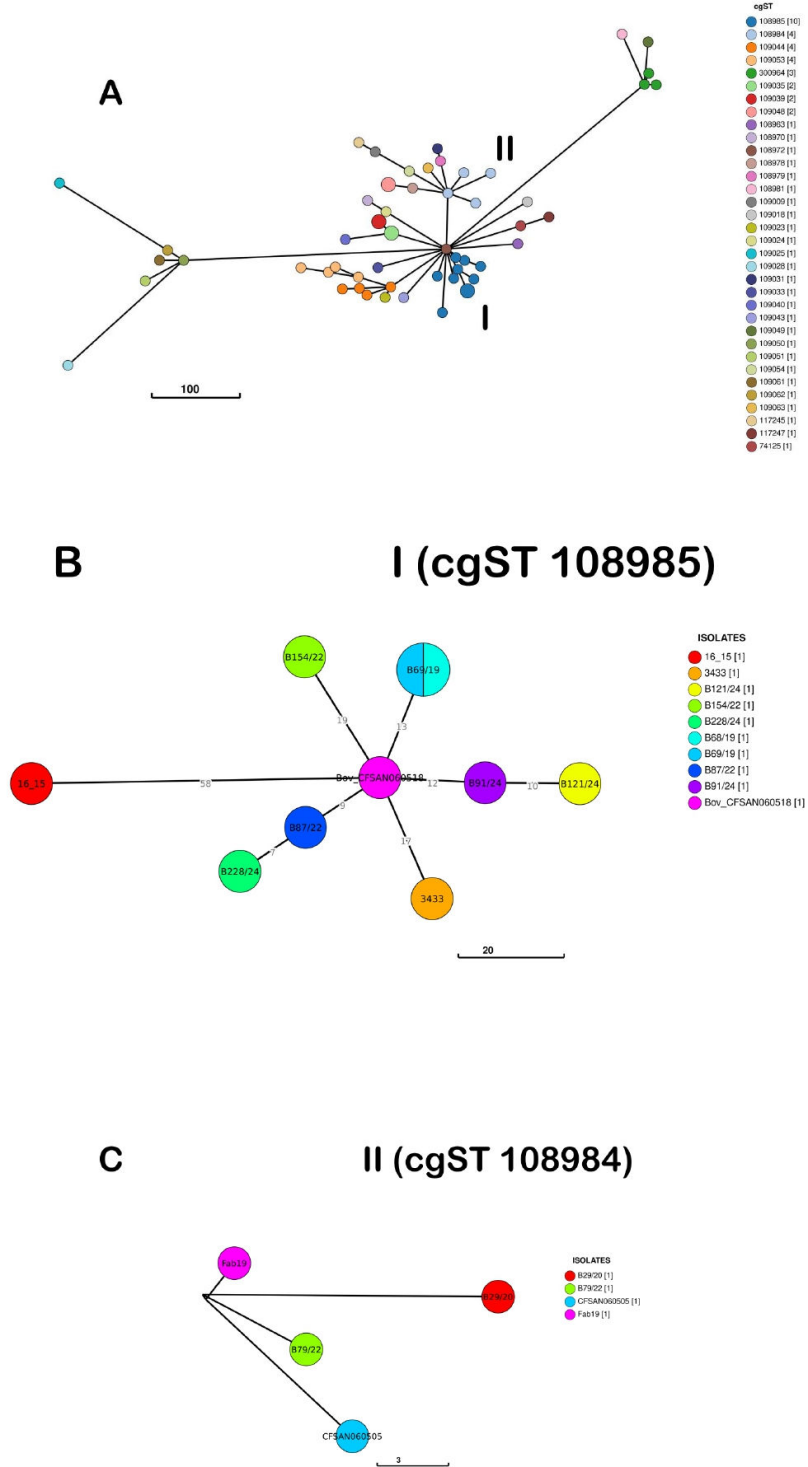
MLST-based minimum spanning tree based on cgMLST allelic profiles of 57 *S.* Dublin isolates. (**A**) Each color represents a different cgST. The numbers (I and II) highlight cgSTs where human strains cluster together with our isolates. (**B**) Subtree B shows cgST 108985, where eight of our isolates, related to seven cases (cases 1, 2, 12, 14, 17, 18, and 19), clustered with a human (16_15) and a bovine (Bov_CFSAN060518) strain from previous studies in Brazil (27). (**C**) Subtree C shows cgST 108984, where three of our isolates, related to three cases (cases 3, 5, and 11), clustered with a human strain (CFSAN060505) from previous studies in Brazil (27).

Among the 57 isolates subjected to cgMLST in the present study, six distinct cgSTs were identified within our isolates (File S6). The strains differed between 0 and 337 alleles, out of 3,002 loci, corresponding to 0%–11.2%, indicating a largely conserved core genome. Strains that were grouped together in the SNP analysis also had the same cgST. Isolates from 10 cases out of the 19 studies were classified into the same cgST as isolates previously recovered from infected humans in Brazil (Fig. 7) (27).

**Fig 8:**
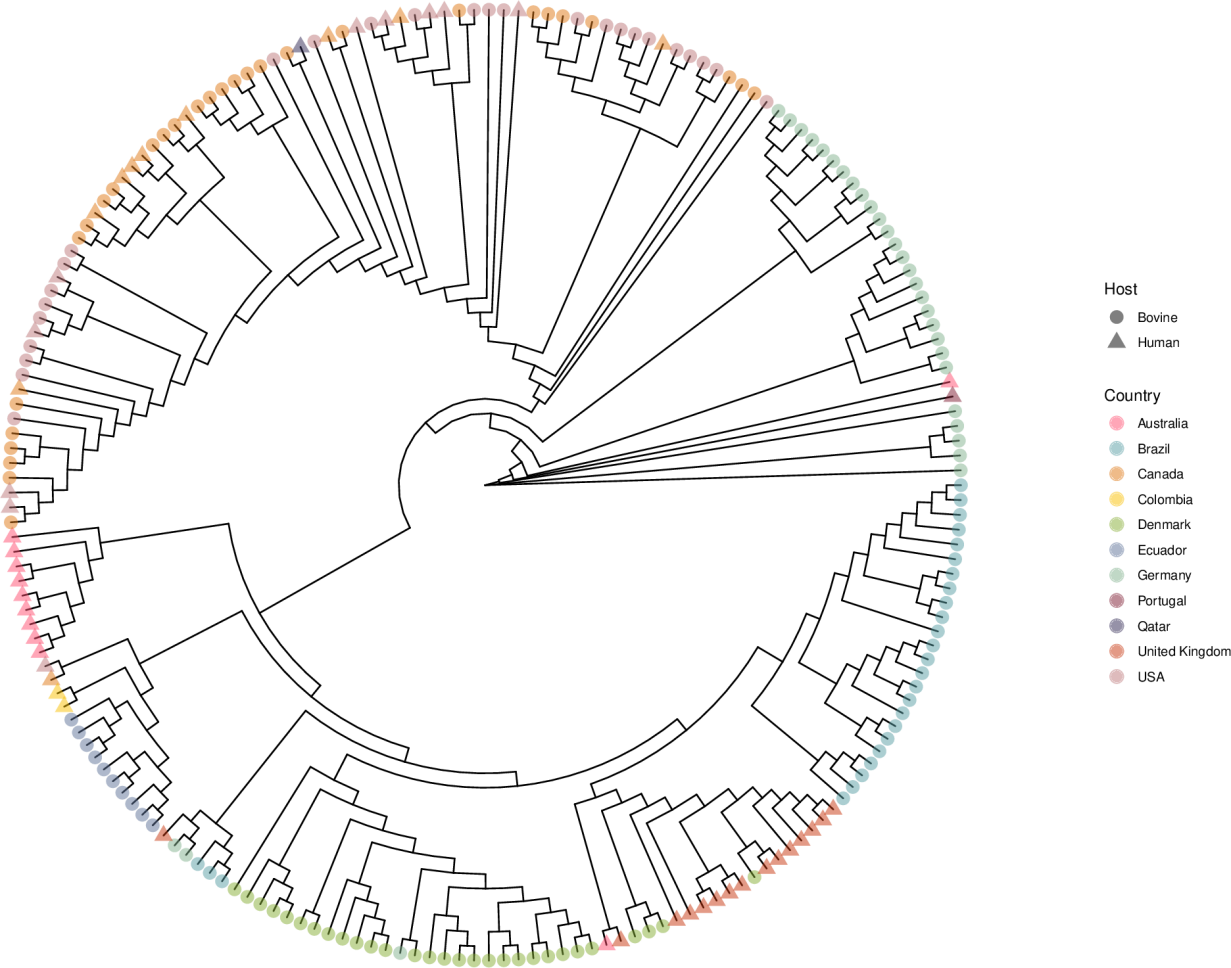
Core genome single-nucleotide polymorphism maximum likelihood phylogeny of *S*. Dublin around the world. Tip shape denotes the host, and tip color denotes the country where the strain was collected.

When compared to *S.* Dublin in other countries (Fig. 8), the strains from this study, as well as the additional five Brazilian strains identified through NCBI, clustered distinctly from other strains. Of the 6,258 annotated genes in this 201-strain global pangenome, 2.2% (*N* = 137) of genes were identified only in the strains from this study. When the additional Brazilian strains are considered, 4.1% (*N* = 259) of genes were unique to Brazilian strains alone. Three strains not included in the primary cluster presumably diverged, evidenced by belonging to a different sequence type, ST3734 (File S7). Additionally, 1% (*N* = 65) of the genes in this data set pangenome were unique to these ST3734 strains. The reason for this divergence is not clear. Nevertheless, it is important to note that, despite these differences, all isolates were classified within the same clonal complex, indicating that they are closely related.

## DISCUSSION

### Clinical-epidemiology features of the outbreaks

This study reports confirmed outbreaks of *S*. Dublin in 19 cattle herds across Brazil. To the best of our knowledge, such studies are rare in Brazil, with only one previous investigation that addressed the clinical and epidemiological aspects of a single outbreak caused by this serovar in cattle (12). In this work, we characterized the *S.* Dublin outbreaks by documenting clinical signs observed in affected animals and post-mortem findings. Additionally, we assessed the antimicrobial susceptibility of the isolates and analyzed their antimicrobial determinants and genetic relatedness to strains from both bovine and human infections from previous reports in Brazil. We further contextualized these findings within the broader global population of *S.* Dublin, highlighting the genomic distinctiveness of Brazilian strains.

*S.* Dublin is known to cause disease in cattle of all ages. However, most reports of *S.* Dublin infection involve suckling calves (9, 13, 14, 74, 75). In contrast, most of the affected animals in our study were between 90 and 180 days old. The main hypothesis for this marked difference is the occurrence of tick fever. In more than two-thirds of the cases included in this study, salmonellosis was either preceded or accompanied by a significant increase in cases of anaplasmosis and/or babesiosis, which are endemic diseases in Brazil (76–79). Indeed, several previous studies have reported salmonellosis as an important secondary infection associated with other diseases, such as distemper in wild animals, canine parvovirus, and malaria in humans (80–83). In *Plasmodium* spp. infections in humans, it is widely believed that the predisposition to salmonellosis is primarily associated with immunosuppression induced by the parasite; nevertheless, additional mechanisms have been proposed, including compromised integrity of the intestinal mucosal barrier and conditions conducive to bacterial proliferation (84–87). Similar to *Plasmodium* spp., a protozoan previously identified as a predisposing factor for salmonellosis in humans, *Anaplasma marginale* and *Babesia* spp. induce hemolysis and immunosuppression in cattle, thereby supporting the hypothesis that these pathogens may constitute a risk factor for the occurrence of *S.* Dublin infections (77, 85, 88–92). This hypothesis may also apply to the salmonellosis outbreak observed in production cows in the present study (case 3). In this case, the simultaneous occurrence of trypanosomosis and salmonellosis was documented, further reinforcing a potential role of co-infections in the pathogenesis of *S.* Dublin infections. It is important to note that this hypothesis has been previously proposed in other studies conducted in Brazil, which identified tick fever as a significant risk factor for *S.* Dublin infections (12, 91).

In addition to comorbidities, other factors may also be associated with the occurrence of salmonellosis in calves aged 90 to 180 days in the present study. Previous investigations have identified the weaning and post-weaning periods as critical, characterizing them as high-risk phases for the development of diseases, including bovine respiratory disease (93–95). The decline in passive immunity, which occurs around 3 months of age, coincides with weaning and is accompanied by changes in diet, group housing, vaccination, anthelmintic treatment, and exposure to environments with higher tick challenge—factors that collectively represent significant stressors in the calf’s life (96–98). These conditions are directly associated with the occurrence of bovine tick fever and may also directly contribute to the development of salmonellosis.

In the present study, an outbreak of hospital-acquired *S*. Dublin infection was diagnosed in calves aged 20 to 60 days housed in a veterinary hospital. The outbreak was notable for its high mortality rate (around 30%). It is worth noting that a previous salmonellosis outbreak caused by *S.* Typhimurium had occurred at the same facility (99). Several studies report *Salmonella* spp. as an important nosocomial pathogen in veterinary hospitals (100–102), this being the first report of an outbreak caused by *S.* Dublin. It is well known that animals housed in hospital environments are at risk of infection by *Salmonella* spp. due to multiple factors, such as intestinal microbiota disruption caused by antimicrobial use and proton pump inhibitor administration, immunosuppression, and concurrent diseases (103–105). Additionally, hospitalized patients exhibit increased shedding of *Salmonella* spp., which can easily spread throughout the environment and infect susceptible animals, leading to nosocomial outbreaks in animals and even humans (6, 102, 106–109).

The majority of *S.* Dublin outbreaks documented in the present study were characterized by clinical signs, such as lethargy, hyperthermia with or without respiratory symptoms (dyspnea and coughing), and mucosal jaundice, which are consistent with previous reports (4, 6, 14, 74, 91, 110–112). The high mortality rate, along with common macroscopic lesions such as petechiae on various serous membranes, pulmonary congestion and edema, suppurative pneumonia, and hepatomegaly, also corroborates previous studies (1, 4, 14, 112, 113). The present study also highlights the occurrence of meningitis in one animal during the nosocomial outbreak at a veterinary hospital (case 5), a recognized manifestation of salmonellosis that is considered uncommon, particularly in cases of *S.* Dublin infection (1, 8, 114). Similarly, enteric manifestations caused by *S.* Dublin are also considered a less frequent presentation of the disease (15, 74, 115, 116), having been reported in three cases in the present study (cases 2, 3, and 19).

### Antimicrobial resistance and genetic determinants in *S.* Dublin isolates

Over the past decade, numerous studies have indicated a significant global increase in antimicrobial resistance among *S.* Dublin isolates, with resistance levels frequently exceeding those observed in other *Salmonella* serovars (22, 117–125). In the present study, more than three-quarters of the isolates exhibited resistance to at least one of the tested antimicrobials, and multidrug-resistant isolates were identified in 21.1% of the cases evaluated. This frequency is higher than that reported in studies from European and African countries (29, 126–133). Conversely, no isolates tested positive for extended-spectrum β-lactamases, contrasting with recent findings reported in the United States and Canada (55, 118, 134–136). While the global strain phylogeny presented in this study did not explicitly explore differences in antimicrobial resistance genes, these geographic differences in resistance nonetheless mirror those observed in strain core genomes.

Regarding specific drug resistance, the prevalence of fluoroquinolone-resistant isolates is of particular concern, with more than a quarter of the isolates and 31.6% of the farms (6/19 = 31.6%) showing resistance. This result is considerably higher than those reported in previous studies conducted in Brazil, as well as in several other countries (31, 70, 72, 136, 137). In line with the phenotypic findings, most of the sequenced isolates harbored point mutations in the *gyrA* gene (S83F and S83Y), a recognized mechanism underlying fluoroquinolone resistance in *S*. Dublin, including in Brazil (27, 68, 138). A possible explanation for the high frequency of resistance observed is the widespread use of this class of antimicrobials in therapeutic protocols for treating diarrhea, pneumonia, and anaplasmosis in calves. In the United States, Canada, Australia, and across Europe, the use of fluoroquinolones is restricted in food-producing animals (139–142). In contrast, in Brazil, the Ministry of Agriculture and Livestock only prohibits the use of fluoroquinolones as zootechnical performance-enhancing additives, with no legislation restricting their use in other circumstances (143, 144).

In addition to the selective pressure for fluoroquinolone resistance, a class considered critically important for human medicine (145), their use in food-producing animals has been increasingly restricted due to concerns regarding co-selection and cross-resistance. Evidence indicates that fluoroquinolone exposure facilitates the acquisition of resistance determinants in Enterobacteriaceae, thereby promoting cross-resistance to other antimicrobial classes (146–149). Indeed, in our study, fluoroquinolone-resistant isolates were nearly 12 times more likely to exhibit multidrug resistance, a finding consistent with reports from other studies (70, 150–152). Overall, these findings suggest the need to reassess fluoroquinolone use in Brazilian cattle production systems.

Isolates from three distinct cattle herds (cases 4, 12, and 15) exhibited resistance to amphenicols, along with the presence of the antimicrobial resistance genes (*floR* and/or *cmlA1)*. Notably, respiratory signs are among the primary clinical manifestations of *S*. Dublin infection (1, 7, 9), for which florfenicol is often employed as the empirical first-line treatment (153, 154). Florfenicol is used exclusively in food-producing animals, whereas chloramphenicol is reserved for the treatment of specific bacterial infections in humans, particularly ocular infections, and occasionally as a last-resort option against multidrug-resistant bacteria that remain susceptible to this antibiotic (155, 156). In addition, the use of chloramphenicol in food-producing animals is prohibited in several countries worldwide, including Brazil (157–159). In this context, the detection of resistance genes to this antibiotic is particularly noteworthy, as it suggests a potential role for horizontal gene transfer in the dissemination of resistance (160, 161).

Interestingly, in this study, amphenicol-resistant isolates were associated with a multidrug-resistant profile, a finding previously reported in other studies (12). This association may be attributed to the acquisition of mobile genetic elements, which are well known for their role in bacterial evolution and the dissemination of antimicrobial resistance (162–165). Indeed, in most cases in our study, the genes conferring resistance to amphenicols were plasmid-borne.

The IncFII(S) and IncX1 plasmids were found in all isolates, similar to previous studies that suggest (27, 55, 70, 124, 130, 166) and indicate that these plasmids are related to virulence (70, 72) and multidrug resistance patterns (117, 167). In the present study, however, the IncFII(S) and IncX1 plasmids were not associated with the antimicrobial resistance genes identified in the isolates.

### Genomic characterization and comparative genomics

All strains evaluated in the present study were classified into clonal complex 53 (CC53), reinforcing that isolates of this serotype compose a highly homogeneous population, as previously reported elsewhere (124, 168, 169). CC53 has also been reported as the dominant lineage of *Salmonella* Dublin, reflecting its strong host adaptation to cattle and widespread global dissemination (118, 124, 169, 170). These observations are further supported by cgMLST and SNP analyses, which revealed high genetic similarity between our strains and isolates previously recovered from both animals and infected humans.

The vast majority of isolates in this study were classified as ST10, which is considered the predominant sequence type of *S.* Dublin in Europe (29, 30, 71, 128, 169, 171). Three isolates were identified as ST3734 and ST4030, both of which also belong to CC53 and have been previously reported in human and animal isolates from Brazil (27). Interestingly, core genome single-nucleotide polymorphism analysis suggested that geography is a clear driver of strain clustering, wherein strains from the same continent have more similar core genomes than when compared to strains from different continents. For example, strains from Canada and the United States are more closely related than strains from Brazil and the United States. This finding is in accordance with previous studies suggesting that geography plays an important role in shaping *S*. Dublin population structure (55, 134, 170, 172–174) and may be explained, at least in part, by differences in livestock exchange patterns. The dissemination of *S.* Dublin is known to be strongly associated with cattle movement (3, 169, 175–180), which has historically been limited between Brazil and the United States but substantially more intense between the United States and Canada, potentially accounting for the greater genetic similarity observed among North American isolates.

Strains from two farms (cases 8 and 10), approximately 893 km (555 miles) apart, differed by only five SNPs, below the 15 SNPs threshold previously established for assessing potential clonality (23, 71, 72). Analysis of the farms’ history confirmed the movement of animals between these properties within the past 5 years, suggesting the epidemiological link to the infection. Based on genetic similarity and historical information, it is likely that the introduction of this *S.* Dublin strain occurred through the purchase of animals, as animal acquisition is a well-recognized risk factor for the introduction of various infectious diseases, including *S.* Dublin (3, 177, 178, 180–182). Despite the adoption of biosecurity measures to contain the spread of *S.* Dublin in cattle herds, there are still no totally effective methods to prevent its introduction and establishment. Denmark’s experience, with a national eradication program in place since 2008, illustrates this challenge, as the country has reported an increase in the prevalence of positive herds even with continued efforts (183–187).

cgMLST analysis has been widely used as a typing method to accurately infer the genetic relatedness and diversity among *Salmonella* isolates (188–190). In our study, cgMLST analysis revealed high similarity between bovine isolates and strains previously associated with human infections in Brazil, corroborating previous studies (11, 27, 28, 70). The link between *S.* Dublin and humans is apparent when considering global strains as well, with strains from both hosts often clustering together. This finding reinforces the epidemiological link between *S.* Dublin isolates from cattle and human infections. Although the exact transmission routes remain largely unknown, it is believed that transmission may occur through direct contact with cattle or, more commonly, through animal-derived products, such as meat, milk, and dairy products (171, 191–193). Human *S.* Dublin infection is frequently associated with high hospitalization and mortality rates, with recent studies demonstrating an increase in both incidence and severity of cases (22, 23, 194–197). In this context, the similarity between *S.* Dublin isolates from animals and human infections, combined with the presence of multidrug-resistant isolates, is particularly concerning. Given these circumstances, it is imperative that veterinarians comprehend their role in the context of antimicrobial resistance (198, 199), highlighting the importance of One Health approaches in such scenarios.

This study has some limitations. First, the sample size (19 farms and 44 isolates), although representative of three major cattle-producing states in Brazil, was relatively small, and most isolates were obtained from dairy farms. Therefore, extrapolation of these findings to other production systems, regions, or countries should be made with caution. In addition, information on antimicrobial use at the farm level was limited, which restricted a more comprehensive interpretation of the antimicrobial resistance profiles observed among the *S*. Dublin isolates. Future studies should incorporate detailed metadata on antimicrobial consumption and include larger, more geographically diverse collections of isolates from different production systems and regions to strengthen the epidemiological understanding of *S*. Dublin in Brazil.

### Conclusion

This study provides the first characterization of multiple *S*. Dublin outbreaks in Brazilian cattle. Most cases involved animals aged three to six months, which contrasts with previous reports that predominantly describe infections in calves up to 30 days old. The high frequency of multidrug-resistant isolates, including fluoroquinolone-resistant strains, combined with the high genetic similarity between strains from cattle and humans, underscores the need for a One Health approach to mitigate the impact of *S*. Dublin in Brazil.

## SUPPLEMENTAL MATERIAL

File S1 spectrum.02665-25-s0001.xlsx Details of the farms included in the present study.

File S2 spectrum.02665-25-s0002.xlsx Details of the Brazilian strains included in the SNP analysis in the present study.

File S3 spectrum.02665-25-s0003.pdf SNP analysis showing the genetic relationships among S. Dublin isolates using *S. Enteritidis* as an outgroup.

File S4 spectrum.02665-25-s0004.docx Antimicrobial resistance determinants and plasmids in the *S. Dublin* isolates from Brazilian dairy farms.

File S5 spectrum.02665-25-s0005.xlsx Matrix of the SNPs analysis comparing *S. Dublin* isolates from humans and bovine in Brazil.

File S6 spectrum.02665-25-s0006.xlsx cgMLST of the *S. Dublin* isolated from cattle and humans in Brazil.

File S7 spectrum.02665-25-s0007.xlsx Details of the *S. Dublin* isolates included in the cgSNP analysis.

ASM does not own the copyrights to Supplemental Material that may be linked to, or accessed through, an article. The authors have granted ASM a non-exclusive, world-wide license to publish the Supplemental Material files. Please contact the corresponding author directly for reuse.
