# Supplementary material for: Salmonella Dublin outbreaks in Brazilian cattle: clinical-epidemiological aspects, antimicrobial resistance, and comparative genomic analysis
Source: Microbiol Spectr. 2026 Mar 12;14(4):e02665-25. doi: 10.1128/spectrum.02665-25 (PMC13055267; doi:10.1128/spectrum.02665-25)
Supplement: File S3 — SNP analysis showing the genetic relationships among S. Dublin isolates using S. Enteritidis as an outgroup. [file spectrum.02665-25-s0003.pdf]

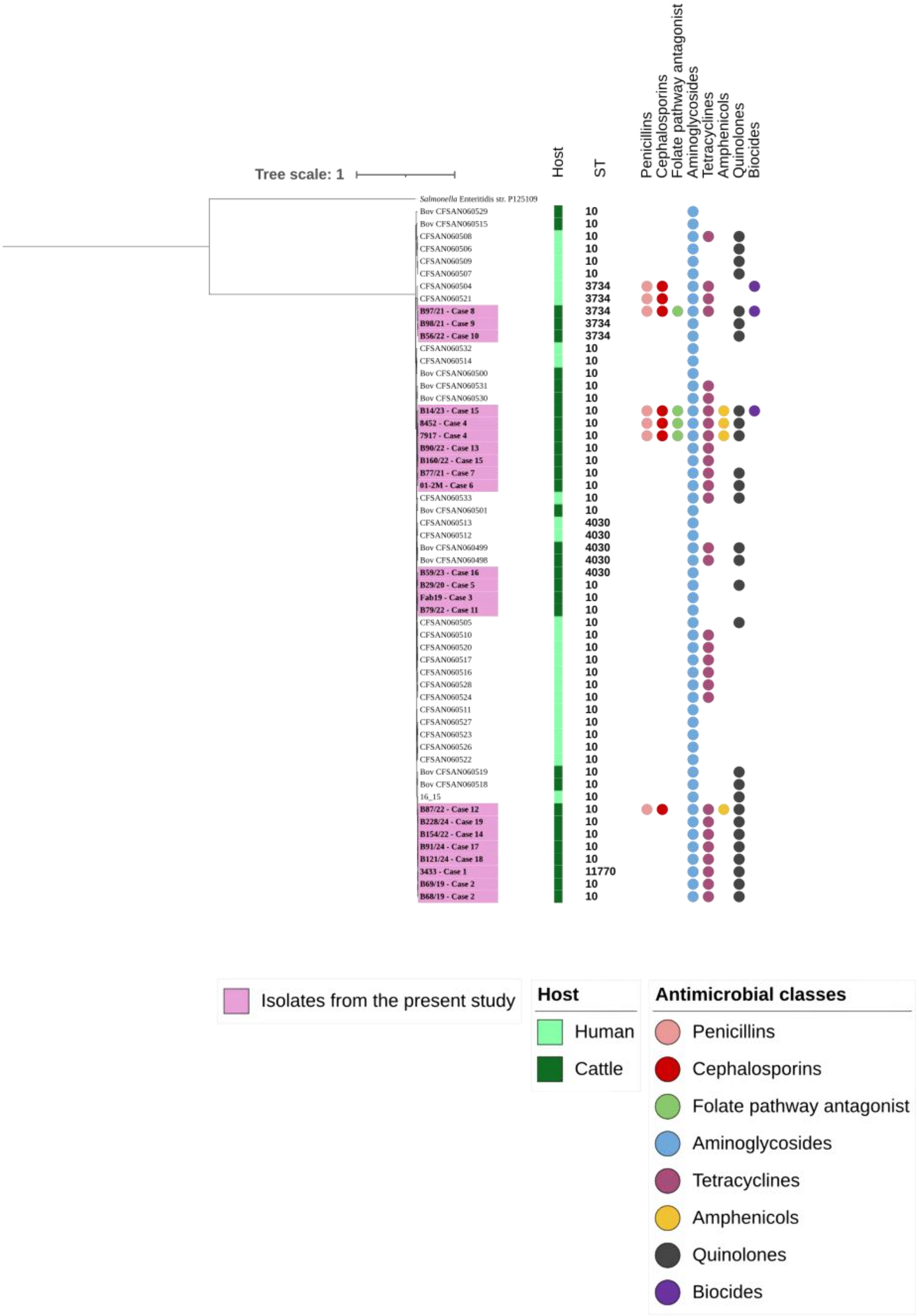


**Supplementary File 3**. Single nucleotide polymorphism (SNP) analysis showing the genetic relationships among *S*. Dublin isolates from cattle and humans. *S*. Dublin isolates from previous studies in Brazil (65) were included for comparison. The phylogenetic tree was inferred using CSIPhylogeny with *S*. Enteritidis as the outgroup reference genome (accession number AM933172). A total of 12,622 high-quality SNPs were used for tree construction, and the tree visualization was generated using iTOL (54).
