# Supplementary material for: Salmonella Dublin outbreaks in Brazilian cattle: clinical-epidemiological aspects, antimicrobial resistance, and comparative genomic analysis
Source: Microbiol Spectr. 2026 Mar 12;14(4):e02665-25. doi: 10.1128/spectrum.02665-25 (PMC13055267; doi:10.1128/spectrum.02665-25)
Supplement: File S4 — Antimicrobial resistance determinants and plasmids in the S. Dublin isolates from Brazilian dairy farms. [file spectrum.02665-25-s0004.docx]

| **Supplementary File 4 – Antimicrobial resistance determinants and plasmids in the *S*. Dublin isolates from Brazilian dairy farms** | | | |  |
| --- | --- | --- | --- | --- |
| **STRAIN ID** | **CASE** | **PLASMIDS** | **AMR DETERMINANTS** |  |
| 3433 | 1 | IncFII(S) | N/D |  |
|  |  | IncX1 | N/D |  |
|  |  | IncW | *tet(A)* |  |
| B68/19 | 2 | IncFII(S) | N/D |  |
|  |  | IncX1 | N/D |  |
|  |  | IncW | *tet(A)* |  |
| B69/19 | 2 | IncFII(S) | N/D |  |
|  |  | IncX1 | N/D |  |
|  |  | IncW | *tet(A)* |  |
| Fab19 | 3 | IncFII(S) | N/D |  |
|  |  | IncX1 | N/D |  |
| 7917 | 4 | IncFIB (AP001918) | *sul3*, *aadA1*, *qacL*, *aadA2b*, *cmlA1* |  |
|  |  | IncFII(S) | N/D |  |
|  |  | IncX1 | N/D |  |
| 8452 | 4 | IncFIB (AP001918) | *sul3*, *aadA1*, *qacL*, *aadA2b*, *cmlA1* |  |
|  |  | IncFII(S) | N/D |  |
|  |  | IncX1 | N/D |  |
| B29/20 | 5 | IncFII(S) | N/D |  |
|  |  | IncX1 | N/D |  |
| 01-2M | 6 | IncFIB (AP001918) | N/D |  |
|  |  | IncFII(S) | N/D |  |
|  |  | IncX1 | N/D |  |
| B77/21 | 7 | IncFIB (AP001918) | N/D |  |
|  |  | IncFII(S) | N/D |  |
|  |  | IncX1 | N/D |  |
| B97/21 | 8 | IncFIB (AP001918) | N/D |  |
|  |  | IncFII(S) | N/D |  |
|  |  | IncX1 | N/D |  |
|  |  | IncFIA | N/D |  |
| B98/21 | 9 | IncFII(S) | N/D |  |
|  |  | IncX1 | N/D |  |
| B56/22 | 10 | IncFII(S) | N/D |  |
|  |  | IncX1 | N/D |  |
| B79/22 | 11 | IncFII(S) | N/D |  |
|  |  | IncX1 | N/D |  |
| B87/22 | 12 | IncFII(S) | N/D |  |
|  |  | IncX1 | N/D |  |
|  |  | IncW | *tet(A)* |  |
|  |  | IncI1-I(Alpha) | *blaTEM-1B* |  |
| B90/22 | 13 | IncFIB (AP001918) | N/D |  |
|  |  | IncFII(S) | N/D |  |
|  |  | IncX1 | N/D |  |
| B154/22 | 14 | IncFII(S) | N/D |  |
|  |  | IncX1 | N/D |  |
|  |  | IncW | *tet(A)* |  |
| B160/22 | 15 | IncFIB (AP001918) | N/D |  |
|  |  | IncFII(S) | N/D |  |
|  |  | IncX1 | N/D |  |
| B14/23 | 15 | IncFIB (AP001918) | N/D |  |
|  |  | IncFII(S) | N/D |  |
|  |  | IncX1 | N/D |  |
|  |  | IncI1-I(Alpha) | *sul2*, *aph(6)-Id*, *floR*, aph*(3'')-Ib* |  |
| B59/23 | 16 | IncFII(S) | N/D |  |
|  |  | IncX1 | N/D |  |
|  |  | Col440I | N/D |  |
| B91/24 | 17 | IncFIB (AP001918) | N/D |  |
|  |  | IncFII(S) | N/D |  |
|  |  | IncX1 | N/D |  |
|  |  | IncW | *tet(A)* |  |
| B121/24 | 18 | IncFII(S) | N/D |  |
|  |  | IncX1 | N/D |  |
|  |  | IncW | *tet(A)* |  |
| B228/24 | 19 | IncFII(S) | N/D |  |
|  |  | IncX1 | N/D |  |
|  |  | IncW | *tet(A)* |  |

N/D – Not detected
